# Ganglioside containing nano-proteoliposome dampens myeloid-derived suppressor cells function and ability to cross-present tumor antigens: a new approach to recover CTL function on tumor bearing individuals

**DOI:** 10.1186/2051-1426-1-S1-P225

**Published:** 2013-11-07

**Authors:** Audry Fernández, Liliana Oliver, Maura Rábade-Chediak, Rydell Alvarez, Arletty Hernández, Leslie Pérez, Luis E Fernández, Circe Mesa

**Affiliations:** 1 Center of Molecular Immunology, Havana, Cuba

## 

A perturbation of normal myelopoiesis, as a consequence of tumor growth and development; leads to the proliferation and accumulation of myeloid derived suppressor cells (MDSC), which impair T-cell functions reinforcing tolerance to tumor antigens (TA). For these reasons it is increasingly clear that successful cancer immunotherapy will require limiting the immunosuppressive effects of these populations. Very small size proteoliposome (VSSP) are nanoparticles consisting of natural outer membranes vesicles derived from *Neisseria meningitidis* with the GM3 ganglioside hydrophobically incorporated. VSSP is used as adjuvant of many therapeutic cancer vaccines currently under investigation in different clinical trials. In this work, we demonstrate that, different from other immune-modulators, VSSP protects and/or recovers the impaired-antigen specific CTL responses, generally observed on tumor bearing (TB) mice. This important feature of VSSP is related to its capacity to reduce the suppression produced by tumor induced-MDSC (tiMDSC). Additionally, TB mice inoculated with VSSP displayed lower percentage of regulatory T cells and antigen-specific CD8+ T cells do not showed the classical CD3ζ chain down-modulation. This last could be associated with the capacity of VSSP to inhibit the up-regulation of ARG1 and NOS2 gene expression on tiMDSC. Based on these results, we conducted a physician-led clinical trial in which, so far, we have demonstrated that VSSP treatment, of metastatic Renal Cell Carcinoma patients, produces a significant reduction of granulocytic MDSC with the subsequent impact on T cells functionality, including, the recovery of CD3ζ chain expression and IFNγ production. Looking deep into the mechanism by which VSSP impaired MDSC functions we demonstrated that VSSP treatment *in vitro* and *in vivo* was sufficient to differentiate MDSC into APCs expressing the co-stimulatory molecules CD40 and CD86, with the consequent loss of the suppressive function. Additionally, we demonstrated that *in vivo* administration of VSSP to TB mice abrogated TA cross-presentation by splenic MDSC, essential mechanism by which tumor specific tolerance is produced. Moreover, TB mice vaccinated with a TA mixed with polyinosinic-polycytidylic acid do not avoid its cross-presentation by tiMDSC, supporting tolerance of specific CTLs. Surprisingly, in this setting, the additional treatment with VSSP revert these negative effects. Altogether, these capacity of VSSP to modified TA presentation and suppression mediated by tiMDSC, could explain the protection of CTL response achieved in TB mice.

